# Religious Socialization and Moral Development: How Similar? How Different?

**DOI:** 10.1093/geroni/igaa057.2269

**Published:** 2020-12-16

**Authors:** Linda George

**Affiliations:** Duke University, Durham, North Carolina, United States

## Abstract

Among Vern Bengtson’s contributions to research on aging, religion, and the family was the finding of strong patterns of intergenerational religious socialization within families. Professor Bengtson and colleagues published at least one book and a dozen journal articles documenting the strong evidence of intergenerational religious socialization, although they also documented specific variations of this general pattern. More recently, social scientists in the culture and cognition tradition have focused on moral development. Most of this research is based on studies of adolescents and emerging adults and concludes that families influence morality at these life stages, but that the effects of peers are even stronger. Some of this research explicitly links morality to religion; most does not. This paper compares research on religious socialization and morality, focusing on similarities and differences in findings, the potential of each tradition to inform and advance the other, and how that can be accomplished. Part of a symposium sponsored by the Religion, Spirituality and Aging Interest Group.

